# A Grip Strength Estimation Method Using a Novel Flexible Sensor under Different Wrist Angles

**DOI:** 10.3390/s22052002

**Published:** 2022-03-04

**Authors:** Yina Wang, Liwei Zheng, Junyou Yang, Shuoyu Wang

**Affiliations:** 1 School of Electrical Engineering, Shenyang University of Technology, Shenyang 110870, China; zhengliwei@smail.sut.edu.cn (L.Z.); junyouyang@sut.edu.cn (J.Y.); 2 Department of Intelligent Mechanical Systems Engineering, Kochi University of Technology, Kami 7828502, Japan; wang.shuoyu@kochi-tech.ac.jp

**Keywords:** deformation sensor, flexible grip, muscle model, strength estimation

## Abstract

It is a considerable challenge to realize the accurate, continuous detection of handgrip strength due to its complexity and uncertainty. To address this issue, a novel grip strength estimation method oriented toward the multi-wrist angle based on the development of a flexible deformation sensor is proposed. The flexible deformation sensor consists of a foaming sponge, a Hall sensor, an LED, and photoresistors (PRs), which can measure the deformation of muscles with grip strength. When the external deformation squeezes the foaming sponge, its density and light intensity change, which is detected by a light-sensitive resistor. The light-sensitive resistor extended to the internal foaming sponge with illuminance complies with the extrusion of muscle deformation to enable relative muscle deformation measurement. Furthermore, to achieve the speed, accuracy, and continuous detection of grip strength with different wrist angles, a new grip strength-arm muscle model is adopted and a one-dimensional convolutional neural network based on the dynamic window is proposed to recognize wrist joints. Finally, all the experimental results demonstrate that our proposed flexible deformation sensor can accurately detect the muscle deformation of the arm, and the designed muscle model and convolutional neural network can continuously predict hand grip at different wrist angles in real-time.

## 1. Introduction

With global aging, the nonfatal injury rate of people has been increasing [1]. The number of disabled people in the world has reached 1 billion, accounting for 15% of the total population [2]. Physical disability seriously affects disabled people’s daily activity, quality of life, and mental health, especially for patients with upper limb disabilities or amputations, whose daily activity is severely inconvenient despite a healthy body physical performance. Therefore, to guarantee the patient’s quality of life while adding no more burden on the family, the exploitation of artificial limbs is highly desirable. In recent years, prostheses for upper limbs have seen great development and a rapidly increasing amount of research [3,4].

With the development of computer-assisted medical technology, human–machine interface technology [5,6,7] has been widely used in the field of rehabilitation medicine, especially in the field of functional assistance for the disabled [8,9,10]. Human–machine interface technology aims to establish communication between humans and computers by using biological signals of the human body itself. The computer receives commands from human biological signals directly and controls some external devices to complete the corresponding actions. All kinds of human actions are directed by the brain and nervous system, and all kinds of actions are carried out by muscle activities. Therefore, to help the disabled limbs recover their functions, the people hope that the disabled limbs can be controlled by their subjective wishes. Researchers have decoded the surface electromyograph (sEMG) signal [11,12] and EEG [13] signals of disabled patients to obtain the intention of the patients.

Li et al. [14] extracted the sEMG signal of the forearm muscle of the human hand, and using the support vector machine classifier to classify nine hand movements after processing the signal, the recognition rate was 98.64%. Geng et al. [15] introduced the concept of a sEMG composed of a high-density sEMG signal space, proposed a classification scheme of gesture recognition based on a sEMG and convolution neural network, and achieved a 99.0% recognition rate for eight gestures. Bhagwat et al. [16] proposed a feature set extraction and projection method based on the sparse filtering of wavelet packet coefficients. The sEMG signals of 15 different finger movements were classified with a 99.52% accuracy. However, these methods do not take the grip into account when studying the grip of an object by hand, and the grip size is important when the user grabs the object with a manipulator [17,18,19]. For grip estimation, Ma et al. [20] collected sEMG signals in the forearm multi-channel, the grip was collected by a force sensor, and predictive models were built using a gene expression programming algorithm and neural network to predict grip. Wu [21] presented an eight-channel force estimation method based on sEMG signals and a generalized regression neural network. Al Harrach [22] used 8 × 8 high-density sEMG signals and a force model to generate muscle strength. However, because the EMG itself is susceptible to interference, weakness, and nonstationarity [23], it will be interfered with by hair, sweat, and other disturbances in daily use, which will affect the recognition accuracy. Muscle deformation signals have also been applied to some applications in human–computer interaction. Numerous studies have been reported in the literature on decoding hand posture information using muscle deformation. Tamaki [24] identified three types of proximal interphalangeal (PIP) joint angles by detecting small muscle deformations using a photoreflector sensor. Kato [25] realized continuous control of the prosthetic wrist by using the deformation of skin surface muscles. Hosono [26] used an optical muscle deformation sensor to collect muscle deformations on the forearm and achieved gesture recognition based on machine learning. In the above literature, wrist posture was ignored, only considering the size of muscle deformations. When recognizing the hand posture, only the muscle deformations could be obtained, and the grip could not be controlled at different wrist angles. Control of grip strength training at different wrist angles was required for patients to perform more complex movements.

To solve these problems, a new type of deformation sensor is developed, which can detect wrist rotation angle and predict grip strength based on muscle changes. Considering that the grip strength is directly related to the length of muscle contraction, the signal of muscle distortion can directly reflect the grip strength. The shape sensor is not affected by hair and sweat, is comfortable to wear, is low cost, and can accurately detect muscle deformations and wrist angle changes. Considering the relationship between muscle deformation and grip strength, the muscle is modeled using the Voigt muscle model [27], which is based on the Hill-type muscle model [28]. By testing the characteristics of the deformation sensor unit and sensor array, and comparing the measure grip strength of the hand with the estimated grip strength of the model, the feasibility of detecting muscle distortion by the deformation sensor and the accuracy of the muscle model is verified. At the same time, wrist rotation information is collected, and the information in a certain time window is classified and identified by using a time series sampling convolution neural network to realize the estimation of the wrist rotation angle. Based on this sensor, continuous grip estimation can be achieved at different wrist angles. It provides strong theoretical support and technical support for the control of mechanical artificial hands to achieve force feedback control.

The organization of the rest of this paper is as follows. Section 2 introduces the basic structure and measurement principles of the sensor. Section 3 presents the sensor layout, overall system, and signal acquisition, and introduces the muscle model used in the estimation of grip strength and the convolutional neural network for wrist angle detection. In Section 4, two subjects are recruited to verify the grip strength of different wrist joints. Finally, conclusions are drawn in Section 5.

## 2. Development of Flexible Arm Muscle Deformation Sensor

### 2.1. Structure and Principle of the Sensor

**Figure 1:**
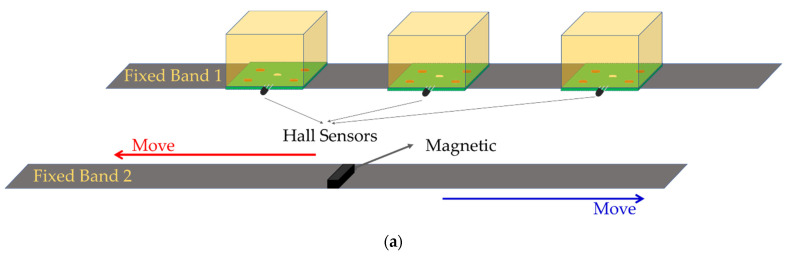

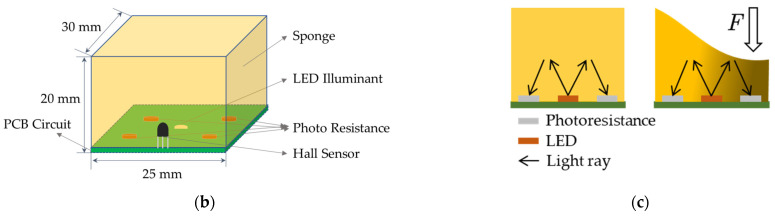
Structure diagram of the sensor: (**a**) overall view of the sensor; (**b**) flexible deformation sensor module; (**c**) flexible deformation sensing principle.

The structure diagram of the developed sensor is shown in Figure 1. As shown in Figure 1a, the sensor consists of three flexible deformation sensor modules and one magnet, which are fixed on two cloth tapes. The distance between the three flexible deformation sensor modules can be adjusted to accommodate different users. Meanwhile, the flexible deformation sensor module consists of a circuit board, an LED illuminant, a Hall sensor, an elastic transparent sponge, and four photoresistors; the basic structure is shown in Figure 1b. While its total size (length × width × height) is 30 mm × 25 mm × 20 mm, the four photoresistors are covered by the light-permeable foaming sponge, and the density of the sponge, which affects its transmittance, is increased or recovered via the squeezing and relaxing from the arm muscle. Moreover, the sensing principle of flexible deformation is shown in Figure 1c. The sensing principle is based on the photoelectric effect, where the photoresistor shows different resistance characteristics under different light intensities, which is affected by the density of the sponge. Therefore, based on the optical properties, the amount of deformation of the arm muscle can be detected by changes in each photoresistance value.

The challenge and special feature are that even though the deformation of the arm muscle with the grip strength is very tiny, our sensor can detect the change in light density to the largest extent as the sponge can perfectly fit into the arm and the special features of its light transmittance. Meanwhile, the photoresistance is also sensitive to light intensity. Therefore, our developed flexible deformation sensor module is sensitive to the deformation of arm muscle. Moreover, there is no trauma to the human body of the sponge, the needed raw materials are easily obtained and have low cost, and the preparation process is simple and has a short cycle. Furthermore, as the magnetic field strength detected by the Hall sensor is related to the relative position between the two bands, the movement of band 2 can be measured by the Hall sensor.

In general, handgrip strength is related to the angle of the wrist. Therefore, we first measured the rotation angle of the wrist by the Hall sensors. Then, we used the flexible deformation sensor modules to detect the deformation of the user’s arm muscle. Finally, we proposed a grip strength-forearm muscle model to detect the grip force based on the wrist rotation angle detected by a timing sequence convolutional neural network (TSCNN).

### 2.2. Circuit Design and Flexible Materials

**Figure 2:**
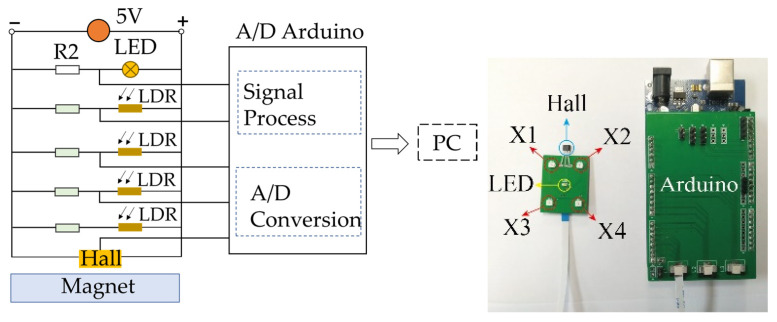
Circuit and signal process of each flexible deformation sensor model.

The detection circuit and signal process of each flexible deformation sensor module are shown in Figure 2. We detected the deformation and finally converted it to the voltage as the sensor output. Specifically, as shown in Figure 2, we utilized the partial voltage principle to detect the photoresistance value by measuring its voltage. Specifically, the photoresistance was connected to a DC circuit in series with constant resistance. When the value of the photoresistance changes, its voltage also changes according to the partial voltage principle. Each flexible deformation sensor module was powered with a 5 V DC power supply, in which the four photoresistances were held in parallel to ensure the voltage consistency, with a resistor R2 in series with a light-emitting diode (LED) functioning in parallel with the four photoresistances to supply illumination for them.

Then, voltage values of the photoresistance were read and the analog-to-digital conversion (A/D) was conducted based on an Arduino microcontroller, which could read 16-way analog voltage signals with a voltage range of 0–5 V, sampling accuracy of 10 bit, and resolution accuracy of 4.88 mV.

**Figure 3:**
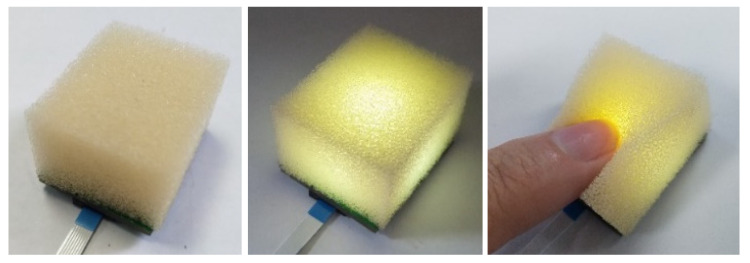
The sponge: its transparency and deformation.

Our developed sensor utilized the sponge as the flexible deformation material, which was particularly sensitive to the slight deformation in the arm muscle and could perfectly fit with the user’s arm, as shown in Figure 3. In addition, the sponge density enlarges or recovers when it is squeezed or relaxed, and the size of density is positively associated with the deformation degree of extrusion. Equally important, as a common material with low cost, the sponge is very comfortable and does not harm the human body. Especially, we chose a sponge with an initial density of 35 d as our selected deformation material by a series of tests to enhance sensor performance to the maximum. The sponge was cropped to a size of 30 mm × 25 mm × 18 mm in length × width × height and stuck to the sensor circuit board by sponge-specific glue. Figure 3 shows the front sides of the fabricated unit sensors, and the status of the sensors with power and with squeezing.

### 2.3. Test of Sensor’s Deformation Detection Properties

**Figure 4:**
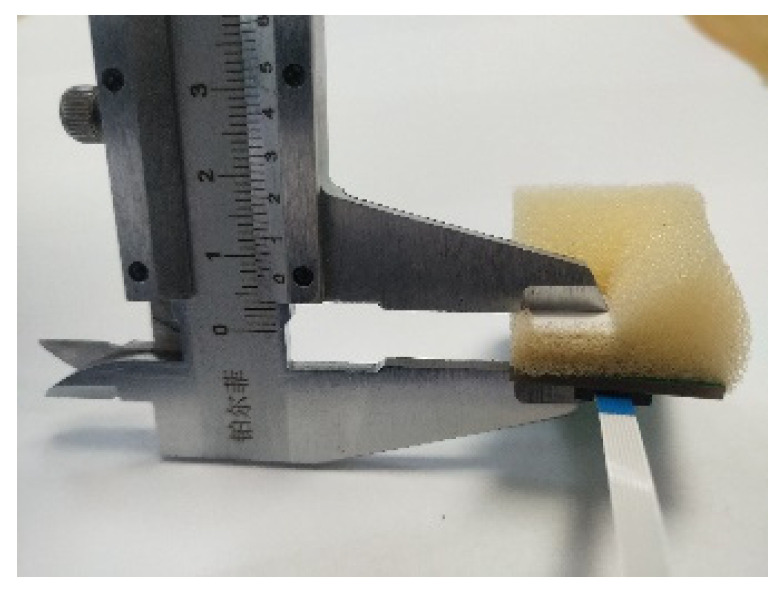
Deformation measurement by the vernier caliper.

In this subsection, we test the detection properties of the developed flexible deformation sensor module oriented to the deformation. As shown in Figure 4, in the experiment, the deformation of the sponge was realized by extruding some designated points while the amount of deformation was measured by a vernier caliper.

Specifically, we conducted two sets of tests. The first set consisted of extruding one point (the X4 point shown in Figure 2) while its deformation changed from 0 to 15 mm with the measurement resolution of 1 mm. The other sets consisted of extruding two points (the X3 and X4 points shown in Figure 2) while its deformation changed from 0 to 15 mm with the measurement resolution of 1 mm. Both tests were repeated 5 times and the voltage outputs of the flexible deformation sensor module were measured by an Arduino Single-Chip Micyoco (SCM).

**Figure 5:**
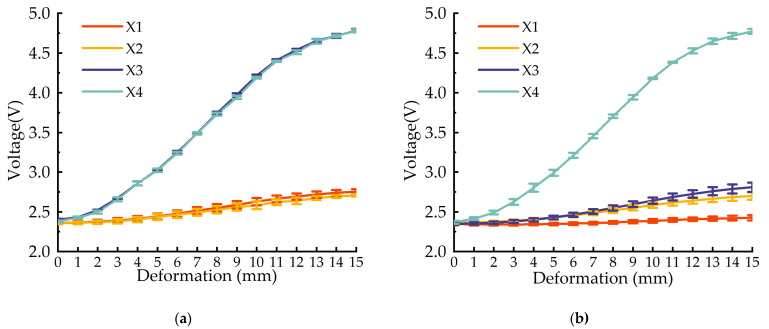
Relationship between deformation and sensor measured values. (**a**) Single point X4 is pressed; (**b**) two points X3 and X4 are pressed.

The experiment results are shown in Figure 5, where Figure 5a shows the results from the X4 point being extruded and Figure 5b shows the results from X3 and X4 points being extruded. As shown in Figure 5, the sensor output voltage responds well to deformation and has a great sensitivity to the deformation. The two sets of experiments show that the sensor has a better consistency, and the repetition accuracy is less than or equal to 1.00%. The deformation characteristics show that there is a nonlinear relationship between the sensor output voltage and deformation quantity, which is determined by the characteristics of the foaming sponge itself.

## 3. Grip Strength Estimation Based on the Flexible Arm Muscle Deformation Sensor

**Figure 6:**
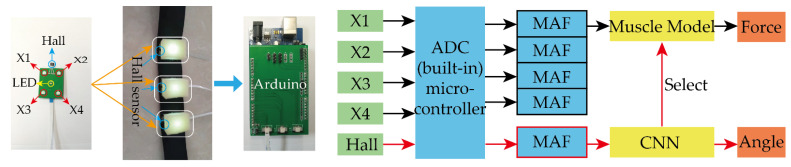
The block diagram of grip strength estimation system.

In this section, the method for grip strength estimation based on the developed flexible arm muscle deformation sensor is introduced. The diagram of the grip strength estimation system is shown in Figure 6. The estimation system contained a single-chip computer module, a PC module, and a power supply device. The resistance value of the photoresistor and Hall signal obtained by the sensor were collected by the ADC built-in single-chip computer. After acquisition, the signal was filtered by a moving average filter (MAF) with a window size of 6. The processed signal was sent to the CNN and muscle model.

As the grip strength is related to the angle of the wrist, as shown in Figure 6, to increase the sensor’s application scenario with an increased estimation accuracy, we predicted the grip force at different wrist angles. Thus, we first detected the wrist angle by the three Hall sensors on the sensor while fixing a magnet on the wrist.

### 3.1. Wrist Angle Estimation

**Figure 7:**
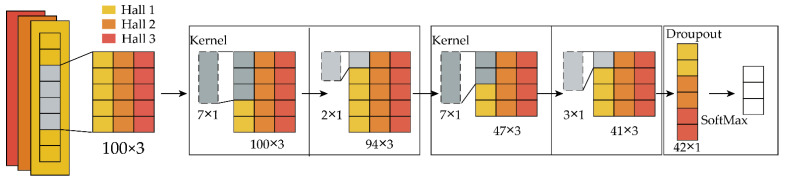
The basic framework of the TSCNN.

**Figure 8:**
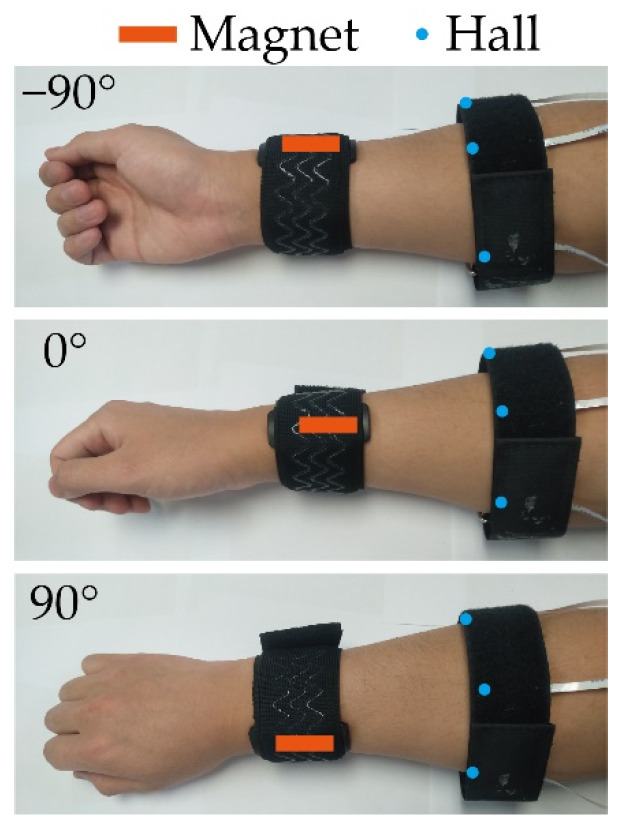
The relative position between magnet and hall at different wrist angles.

In this subsection, a novel wrist angle estimation method is proposed based on the time series one-dimensional convolutional neural network (TSCNN) by Hall sensors. The basic framework of the TSCNN is shown in Figure 7, it has a strong anti-interference ability. The TSCNN can obtain information in one of the three sensor time windows and can still accurately classify Hall sensors when they are disturbed, such as several sudden changes or loss of 100 data in one time window. As, when the wrist rotates, the magnet fixed on the wrist will rotate along with the wrist, the magnetic field at the sensors will change. Figure 8 shows the relative position between the magnet and the Hall at three kinds of wrist twist conditions. We detected the wrist angle using the changing magnetic field measured by Hall sensors based on the TSCNN.

The TSCNN consisted of two main parts: sampling processing in the time dimension and convolutional neural network. The time dimension consisted of the input layer and reconstruction layer, while the convolutional neural network consisted of the convolution layer, maximum pooling layer, and classifier layer. A dynamic window was adopted to sample and stitch the one-dimensional vector data detected by the three sensors, which can expand the valuable feature information of the Hall sensor in the time dimension. The sampling processing sampling network structure mainly included the input layer and the reconstruction layer, as shown in Figure 7.

### 3.2. Grip Strength Estimation

The most challenging issue in the estimation of grip strength is that the generation of handgrip strength of humans is complex. For people without disabilities, the handgrip strength is generated by the simultaneous action of the small arm muscles and the parts of the muscles on the palm. Nevertheless, for wrist amputation patients, the best way to restore grip control is with the help of the lower arm’s muscles.

**Figure 9:**
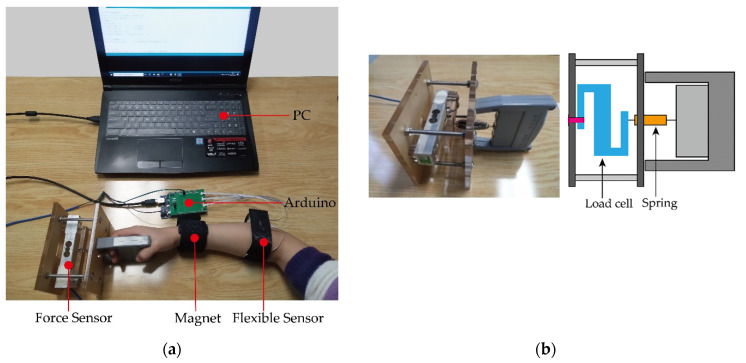
Information acquisition system. (**a**) General diagram of information acquisition system; (**b**) force sensor based on HX711 module.

In our study, to realize the continuous estimation of grip strength, we derived a novel grip strength-forearm muscle model aimed at the healthy human based on the relationship between the forearm muscle’s deformation and grip strength. To derive and validate our derived model, the overall test detection appearance is shown in Figure 9. The designed flexible deformation sensor was fixed on the arm to collect the muscle deformation information of the arm. A magnet was fixed to the wrist that can change its position as the wrist rotates and can be detected by a Hall sensor on the flexible deformation sensor. Handgrip information was obtained using the HX711 force sensor shown in Figure 9b. It can measure the grip strength at different wrist angles. Considering that the study aimed to restore the grip control of the handicapped, the maximum sampling range of the grip meter was 35 N, which was able to accomplish most of the daily tasks such as holding cups, books, and fruits. All information was collected by an Arduino and sent to the PC for information processing.

**Figure 10:**
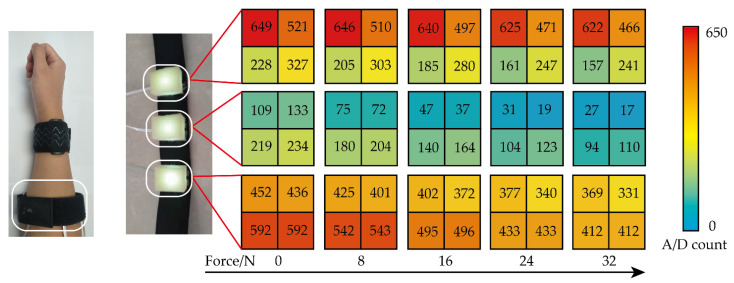
Relationship between signal of sensor and grip strength at one specific wrist angle.

The relationship between deformation information at one specific angle and grip force is shown in Figure 10. It can be seen that there is a certain relationship between grip force and muscle changes on the small arm. The grip force can be estimated by muscle deformation by the grip strength-forearm muscle model.

**Figure 11:**
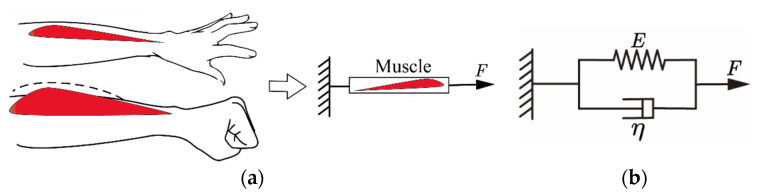
Muscle model. (**a**) Muscle mechanics model; (**b**) Voigt muscle model.

Considering the relationship between muscle deformation and grip strength, the viscoelastic characteristics of the muscle were used as a function of handgrip force and muscle deformation. Therefore, the Voigt muscle model was used to describe the relationship between muscle deformation and grip force, which is based on the Hill muscle model and includes viscous and elastic elements. To apply muscle characteristics to the handgrip, the actual forearm was abstracted into a mechanical model, as shown in Figure 11a. When a person controls the strength of the hand, the arm muscles contract lengthwise, which results in muscle deformations. The skin surface changes as the muscle deforms in the direction of contraction. We assumed that the input muscle deformations of the function and the output grip force of the function correspond to the viscoelastic model of the muscle.

The derived grip strength-forearm muscle model is shown as:

(1) $$F=aX+b\dot{X}+c$$

where *F* denotes the grip strength, ***X*** = [*X*_1_, *X*_2_, …, *X*_12_]^T^ denotes the muscle deformation measured by the deformation sensor, $\dot{X}$ is the derivative of ***X***, ***a*** *=* [*a*_1_, *a*_2_, …, *a*_12_] and ***b*** *=* [*b*_1_, *b*_2_, …, *b*_12_] are the elastic modulus and viscosity parameters, respectively, and *c* are the fitting parameters. The model parameters ***a***, ***b***, and c are very important for the perdition accuracy of the model. To obtain the appropriate model parameters, we utilized the least squares method to perform parameter estimation. The sum of squares of residuals according to the least squares principle is shown as:

(2) $$Q=\sum_{i=1}^{n}{\left(F_{i}-F\right)}^{2}=\sum_{i=1}^{n}{\left(F_{i}-c-\sum_{j=0}^{12}\left(a_{j}X_{ij}+b{\dot{X}}_{ij}\right)\right)}^{2}$$

where *n* = 1000 denotes the number of datasets. The datasets were collected by the experiments in which the subjects gradually force their hands from relaxation to reach maximum grip strength.

## 4. Experiment

In our experiments, to validate the effectiveness of the proposed grip strength sensor, two healthy subjects called A and B were introduced to the experiments. The experimental procedures involving human subjects described in this section were agreed upon by all subjects and conformed to ethical rules.

**Figure 12:**
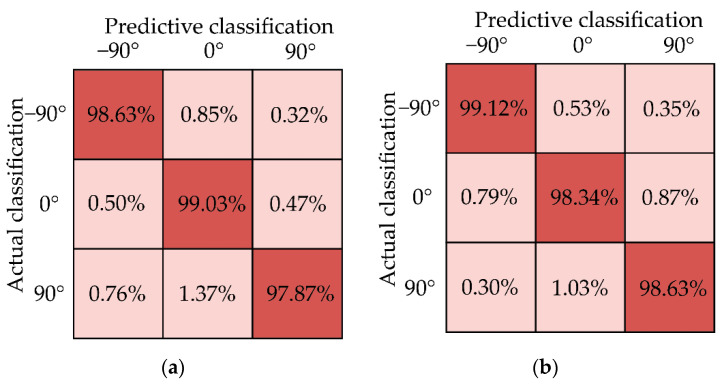
Confusion matrix. (**a**) Confusion matrix of subject A. (**b**) Confusion matrix of subject B.

We first collected the forearm muscle and grip force data at three specific different wrist angles. In detail, subjects held the self-designed grip strength meter in their hands and slowly powered until the maximum range (35 N) of the grip strength meter, rested for 30 s to avoid muscle fatigue, and then repeated the foregoing operation. The experimental process was conducted with the 0° wrist angle, −90° wrist angle, and 90° wrist angle, as shown in Figure 8. Data processing was divided into three steps: (1) data cutting, (2) normalization, and (3) labeling. First, we cut the sample data and then used 200 ms as a time window to splice the data of three Hall sensors. We especially used the min-max standardized formula to normalize the data to [0, 1], namely

(3) $$M=\frac{m-m_{\min}}{m_{\max}-m_{\min}}$$

where M represents the normalized value, m represents the value of sample data, $m_{\min}$ represents the minimum value of one line of data, and $m_{\max}$ represents the maximum value of a row of data. Finally, the dataset was manually added to the angled label and fed into the neural network for training. To verify the effectiveness of the convolutional neural network model, the dataset was trained and tested, and the dataset was divided into the training set and test set in a ratio of 4:1. To clearly show the accuracy of the model in identifying three wrist angles, the identification confusion matrix was drawn, as shown in Figure 12. It can be seen that the recognition accuracy rate of the model for three wrist angles is above 97%. It can be seen that the model established for specific people has a high recognition rate and usability.

Next, we determined the parameters of the grip strength-forearm muscle models in different wrist angles. The least-squares method was used to fit different angles. In this paper, three different wrist angles were introduced. After determining the parameters of the model, the participants were asked to exert force at different wrist angles, and the estimated and measured grip were recorded, as well as the confusion matrix of wrist angles.

**Figure 13:**
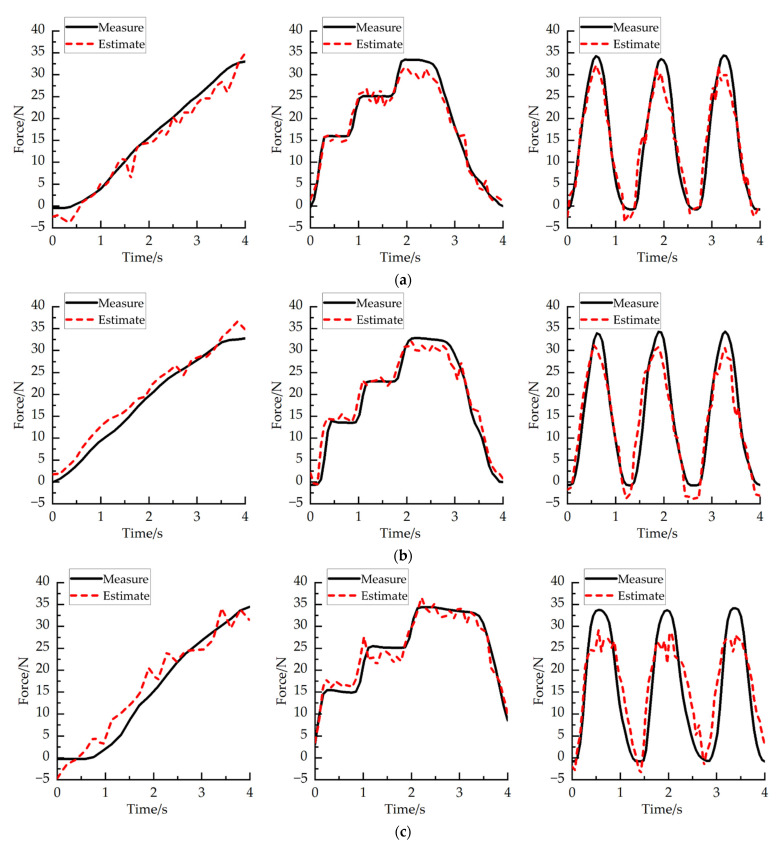
Relationship between measured grip strength and estimated grip strength of subject A. (**a**) The relationship between the measured grip strength and the estimated grip strength when the wrist angle of subject A is 90°. (**b**) The relationship between the measured grip strength and the estimated grip strength when the wrist angle of subject A is 0°. (**c**) The relationship between the measured grip strength and the estimated grip strength when the wrist angle of subject A is −90°.

**Figure 14:**
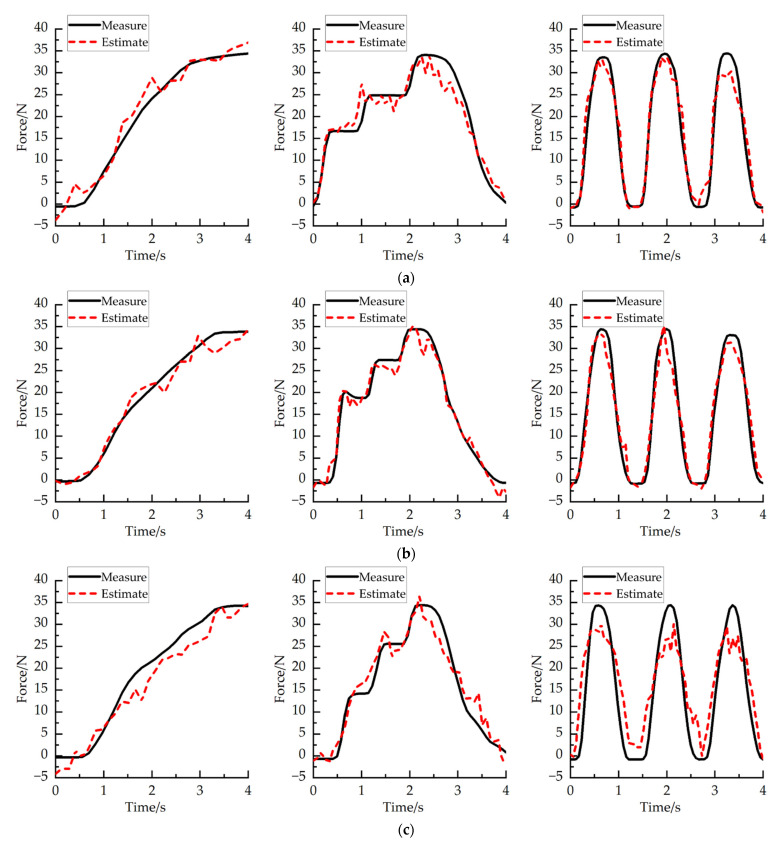
Relationship between measured grip strength and estimated grip strength of subject B. (**a**) The relationship between the measured grip strength and the estimated grip strength when the wrist angle of subject B is 90°. (**b**) The relationship between the measured grip strength and the estimated grip strength when the wrist angle of subject B is 0°. (**c**) The relationship between the measured grip strength and the estimated grip strength when the wrist angle of subject B is −90°.

**Figure 15:**
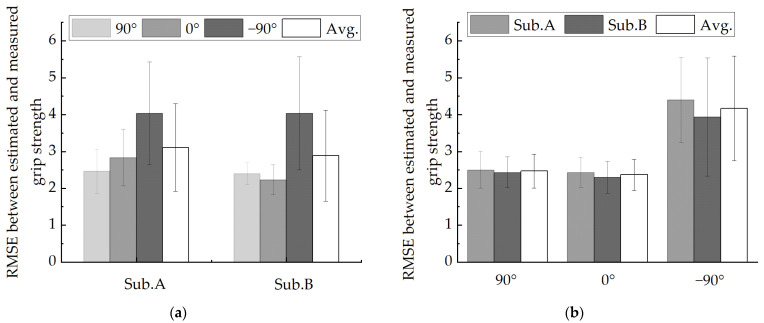
RMSE between the measured and estimated force of the model. (**a**) RMSE between measured and estimated values of participants at three different wrist angles. (**b**) RMSE between estimated and measured values of each participant at different angles.

In order to evaluate the validity of the model, the root-mean-square error (RMSE) between the estimated grip force and the measured grip force was used as the criterion, and the calculation formula is as follows:

(4) $$\mathrm{RMSE}=\sqrt{\sum_{i=1}^{n}\frac{{\left(F_{p}-F_{m}\right)}^{2}}{n}}$$

where $F_{m}$ is measured grip strength, $F_{p}$ is estimated grip strength, and n is the sampled times. In this study, RMSE analysis was performed on each subject at different wrist angles. Figure 13 and Figure 14 show the time series of measuring and estimating the grip strength of two subjects in the experiment under three different wrist joints. The average RMSE between the measured and estimated grip strength of subject A is 3.11 ± 1.19 N, and that of subject B is 2.89 ± 1.24 N. Figure 15a shows the RMSE of two subjects under different wrist joints. Figure 15b shows the RMSE of two subjects at different joint angles. The time series of grip strength shows that the estimated grip strength is in good agreement with the measured grip strength. When the angle changes rather than remains stable, the estimation is more accurate. In the experiment, it is found that the RMSE of the wrist angle is small when the wrist joint is 90° (RMSE = 2.467 ± 0.456) and 0° (RMSE = 2.367 ± 0.423), and it increases when the wrist joint is −90° (RMSE = 4.167 ± 1.39257). We think that the reason for this is that when the wrist angle is −90°, the arm rotation angle of the human body is large, which will make the human hand feel uncomfortable to a certain extent when exerting force, which will lead to a certain deviation in the result.

## 5. Discussion

In this study, a flexible deformation sensor is designed to detect the muscle deformation of the human arm, to predict the size of grip force, and to detect different wrist angles through the sensor’s built-in Hall sensor, to predict the size of grip force at different wrist angles. The results show that the correlation coefficient between grip strength and muscle deformation is 0.96 on average. To some extent, the range achieved can be compared to those reported by others. For example, Gurram [29] and others used power curve regression analysis to find that the correlation coefficient between grip strength and flexor EMG was between 0.91 and 0.99. Keir [30] and Hoozemans [31] reported correlation coefficients between sEMG and grip strength of 0.90 and 0.96, respectively. Ernest et al. [32] used intramuscular electromyography to predict grip strength in 10 subjects over a grip strength range of 0–25/50 N. The report showed that the linear correlation coefficient between iEMG characteristics and grip strength was 0.9, with no significant difference between sEMG and grip strength.

These studies all used multichannel EMG signals, which showed a high correlation between EMG and grip strength. However, the EMG signal itself was susceptible to interference and nonstationarity. There was a high degree of complexity in EMG signal processing. Most of the above studies were performed at a single wrist angle. This study predicts grip strength at three different wrist angles and has a high correlation at three different wrist angles. The results show that grip estimation accuracy is affected to a certain extent at different wrist angles. For example, when the wrist angle is −90°, both subjects show large estimation errors.

Our research also has limitations: the hardware system is still rough and there are many possibilities for improvement, especially in dealing with the interference of external ambient light and the suitability for wear. Muscle models may be optimized later to find a more appropriate one. In addition, we only analyze the tests performed on the arm of the patient in a fixed posture, and then consider the estimations of grip strength in the free state of the arm.

In this study, three different wrist joints correspond to three different sets of parameters. More wrist angle grip data are difficult to obtain and machine learning seems to have a solution to this problem. In future work, we will use machine learning to obtain muscle model parameters at different wrist angles, and we will train with the calculated wrist angle and muscle deformation information as input and grip strength as output. It is difficult for traditional methods to estimate the displacement of muscles at multiple wrist angles, and machine learning can solve this problem. However, the prerequisite for solving this problem is that the wrist angle is still discrete. Estimating the grip force corresponding to a fully continuous wrist joint is still difficult and may require considerable calculation.

## 6. Conclusions

A handgrip estimation system based on a flexible deformation sensor is proposed in this paper. The system can be used to estimate the handgrip strength at different wrist angles with good accuracy and stability. The principle of sensor fabrication, information collection and processing, and muscle model are studied. The characteristics of the flexible deformation sensor are analyzed. The experiments show that the single-point and multi-point measurement errors are small. Meanwhile, a convolution neural network is introduced to identify wrist angle and a muscle model is introduced as a grip estimation function to better describe the nonlinear relationship of human muscle deformation. The experimental results verify the accuracy and real-time of the designed flexible deformation sensor and the grip force estimation based on the muscle model. It can be prepared for subsequent manipulator control.

## Data Availability

Data sharing is not applicable to this article.
